# Supplementary material for: Globally correlated conformational entropy underlies positive and negative cooperativity in a kinase’s enzymatic cycle
Source: Nat Commun. 2019 Feb 18;10:799. doi: 10.1038/s41467-019-08655-7 (PMC6379427; doi:10.1038/s41467-019-08655-7)
Supplement: Supplementary file 1 — Supplementary Information [file 41467_2019_8655_MOESM1_ESM.pdf]

Supporting Information

Globally Correlated Conformational Entropy Underlies Positive and Negative Cooperativity in a Kinase's Enzymatic Cycle

Yingjie Wang,[1,2#] Manu V.S.,[2#] Jonggul Kim[1,2#†], Geoffrey Li, [1‡] Lalima G. Ahuja, [3] Philip Aoto, [3] Susan S. Taylor, [3] and Gianluigi Veglia[1,2]*

[1]Department of Chemistry and [2]Biochemistry, Molecular Biology, and Biophysics, University of Minnesota, Minneapolis, MN 55455
[3]Department of Chemistry and Biochemistry, and Pharmacology University of California at San Diego, La Jolla, CA 92093

# These authors contributed equally to the manuscript

† Present address: Department of Biophysics and Howard Hughes Medical Institute, University of Texas Southwestern Medical Center, Dallas, TX 75390, USA.

‡ Present address: Department of Biochemistry, Vanderbilt University School of Medicine, Nashville, Tennessee 37240, United States.

***Corresponding Author**

G.V. (e-mail: vegli001@umn.edu)

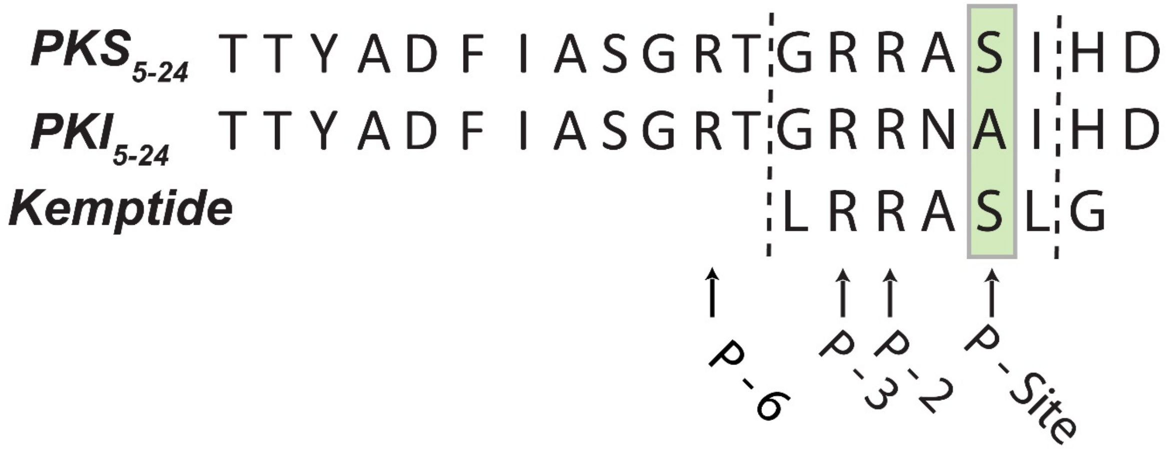

Supplementary Figure 1: Primary sequences of $PKS_{5\text{-}24}$, $PKI_{5\text{-}24}$, and the recognition sequence Kemptide.

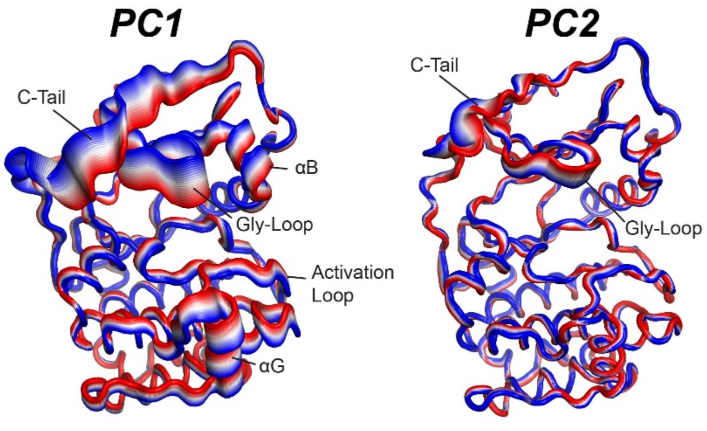

Supplementary Figure 2: Principal Components for the major conformational states as identified by the X-ray structures along the enzymatic pathway, and the color codes correspond to the projection along the principle components in Fig. 1, where the red and blue colors indicate the minimum and maximum values, respectively.

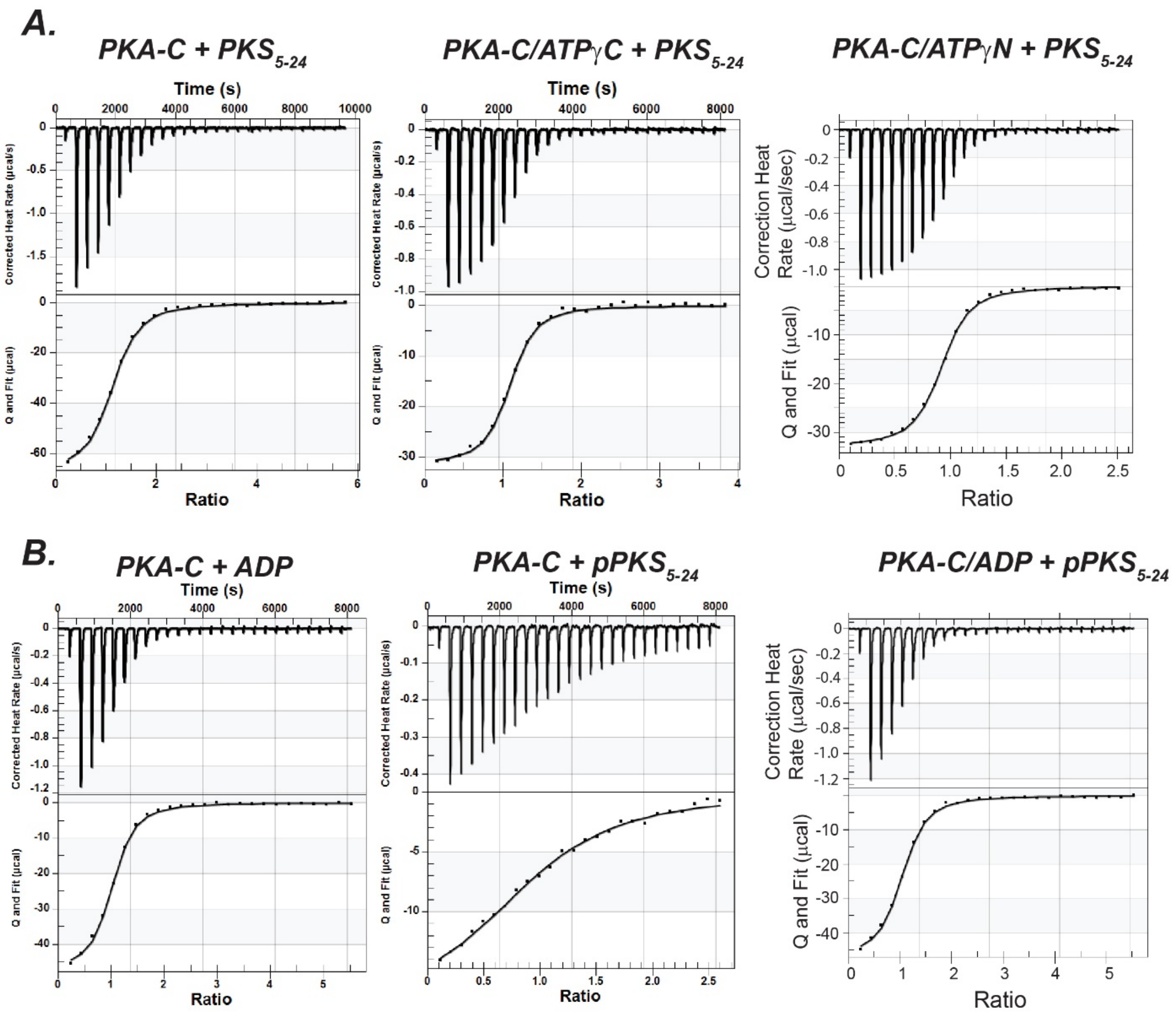

Supplementary Figure 3:

A. ITC isotherms of $PKS_{5\text{-}24}$ binding to Apo, ATPγC and ATPγN saturated PKA-C. **B.** ITC isotherm of ADP and $pPKS_{5\text{-}24}$ binding to Apo PKA-C, as well as ITC isotherms of $pPKS_{5\text{-}24}$ binding to ADP saturated PKA-C

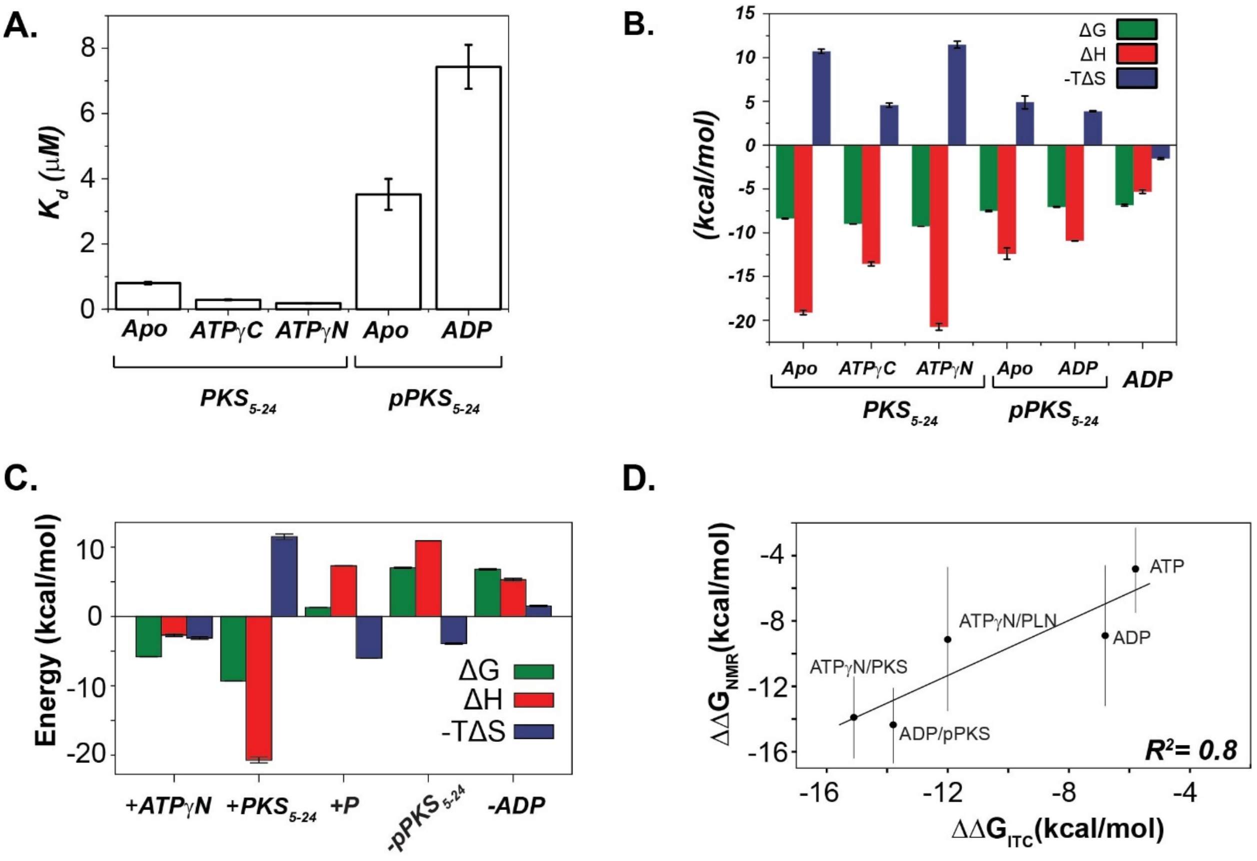

Supplementary Figure 4: Summary of the thermodynamics of $PKS_{5\text{-}24}$, $pPKS_{5\text{-}24}$ and ADP binding to PKA-C. **A.** Binding affinity of $PKS_{5\text{-}24}$, $pPKS_{5\text{-}24}$ to apo and nucleotide-saturated PKA-C (n=3). **B**. Thermodynamics of $PKS_{5\text{-}24}$, $pPKS_{5\text{-}24}$ and ADP binding to different forms of PKA-C. **C**. Thermodynamics of each step over the catalytic cycle. **D**. Linear correlation of Changes in the free energy of binding (ΔΔG) obtained from the CONCISE analysis against the ΔΔG determined by isothermal titration calorimetric (ITC) measurements (mean ± SD, n=3), with R^2 = 0.8. The CONCISE data are obtained from probability density distributions of the NMR chemical shifts for the ILV methyl groups of PKA-C, where the error bar reports the 90% confidence interval.

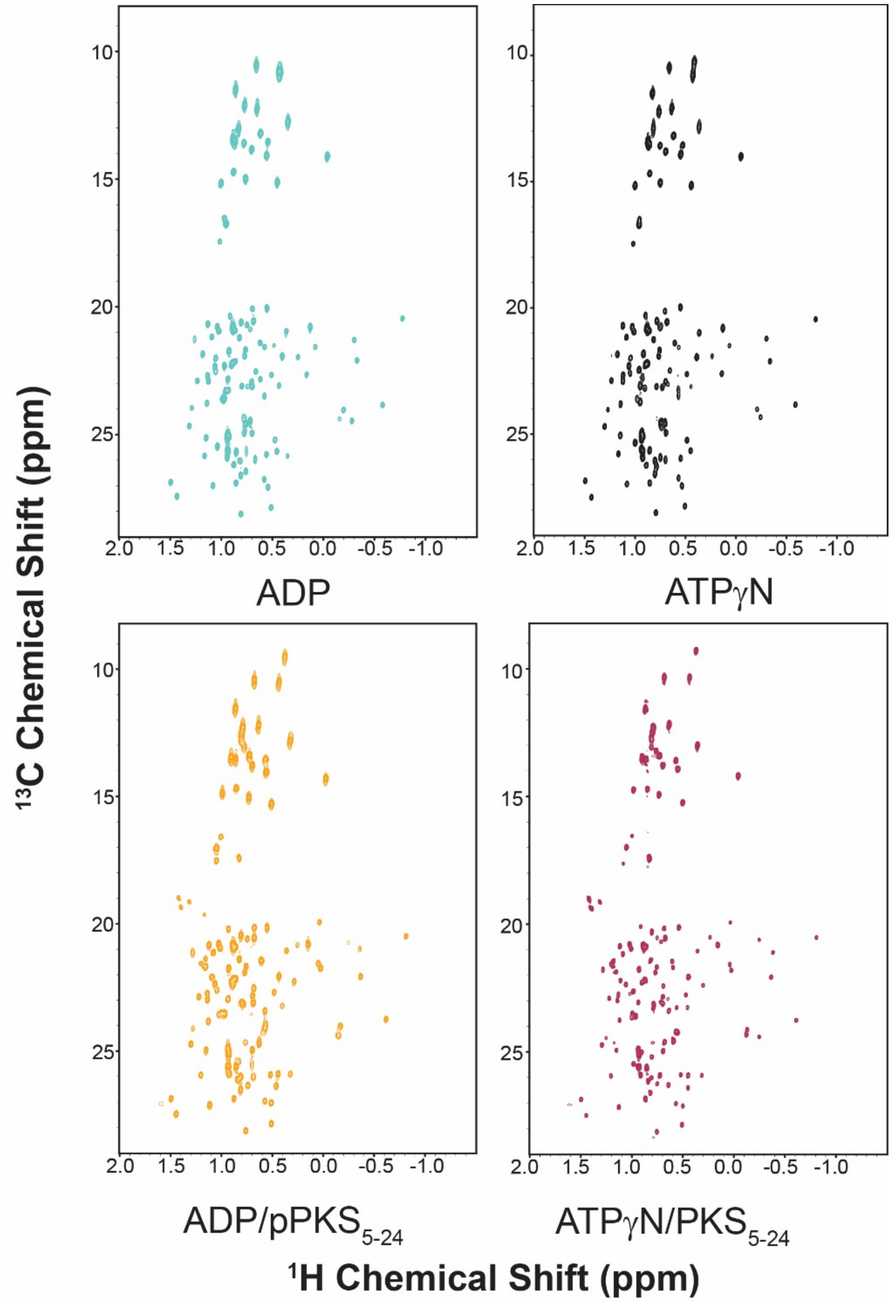

Supplementary Figure 5: ^{1}H-^{13}C methyl-TROSY spectra of ligand bound forms of PKA-C; PKA-C/ADP (Turquoise), PKA-C/ATPγN/60mM Mg^{2+} (black), PKA-C/ADP/pPKS$_{5-24}$ (orange), and PKA-C/ATPγN/PKS$_{5-24}$ (maroon).

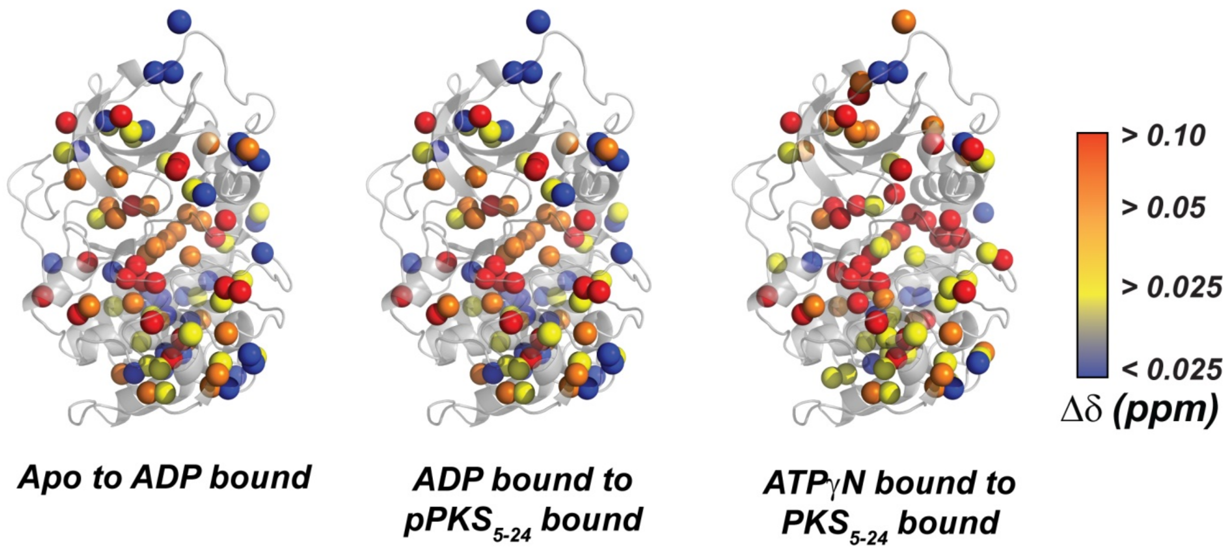

Supplementary Figure 6: Chemical shift perturbation upon ADP (left), $pPKS_{5\text{-}24}$ (middle), or $PKS_{5\text{-}24}$ (right) binding to PKA-C mapped onto the crystal structure of PKA-C (PDB: 1ATP).

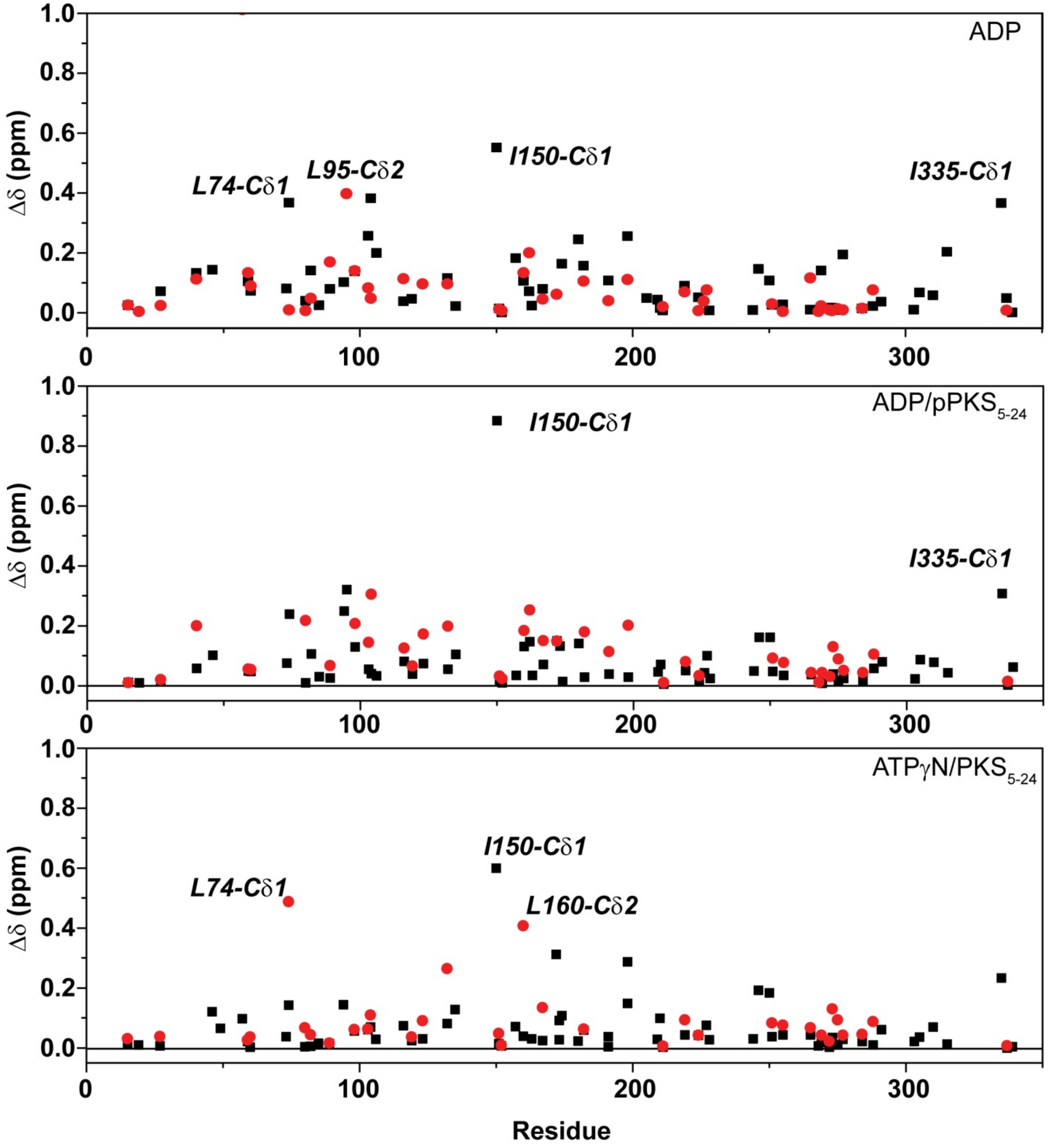

Supplementary Figure 7: Chemical shift perturbation of methyl groups upon binding of ADP to the apo enzyme or peptide ($pPKS_{5\text{-}24}$, $PKS_{5\text{-}24}$) to the nucleotide bound form of PKA-C.

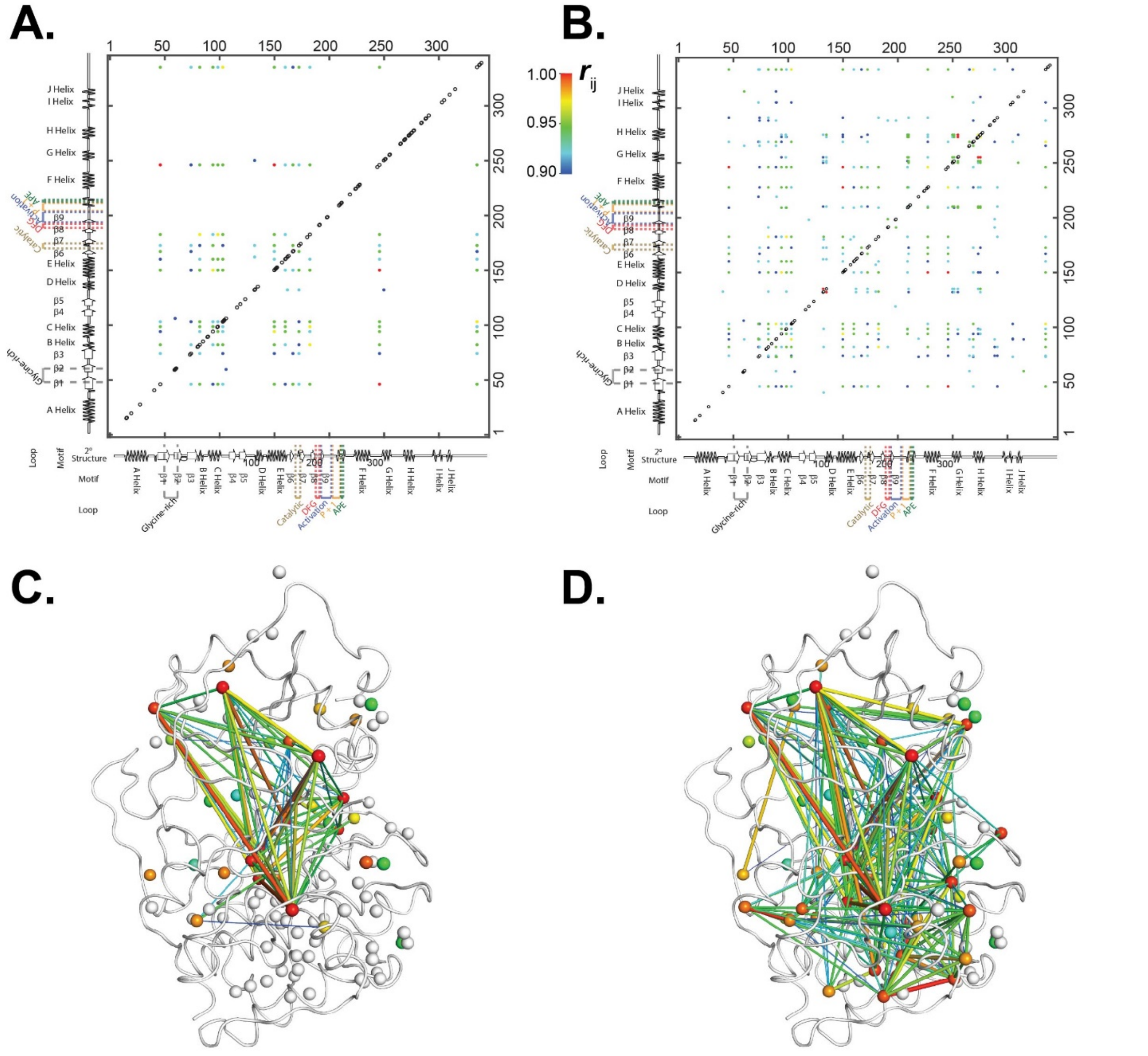

Supplementary Figure 8: Top: CHESCA plot showing the network of allosteric changes in PKA-C upon binding of ATPγN. CHESCA plots with r_{ij} > 0.9 and vector distance cutoff 0.1 (A) and 0.05 (B), respectively. C and D corresponding web plots showing the highest correlated residues in the transition from apo to nucleotide-bound state.

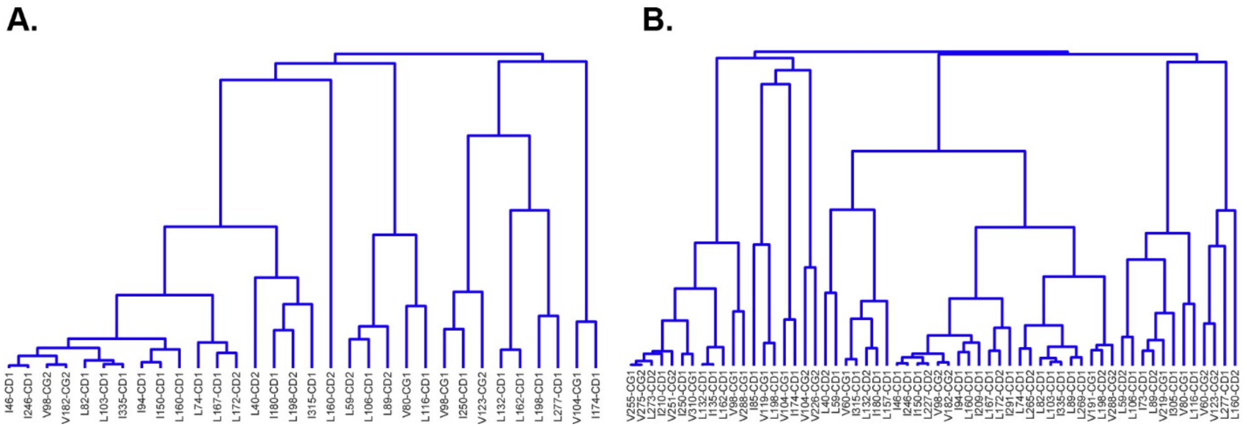

Supplementary Figure 9: Dendrograms from hierarchical clustering obtained from the CHESCA analysis (A) uses higher vector distance cutoff of 0.1 in the correlation matrix (Supplementary Figure 8A) and (B) uses a lower vector distance cutoff of 0.05 (Supplementary Figure 8B), describing the clustering of methyl groups chemical shifts that move through correlated linear trajectories with ADP, ATPγN with high Mg^{2+}, ADP and $pPKS_{5\text{-}24}$, and ATPγN and $PKS_{5\text{-}24}$. Note that under different cutoffs, the majority of residues for most of the states fall under a single group, showing that the entire enzyme allosterically moves between the open and closed states except for the ADP bound state where the residues are split off into smaller groups.

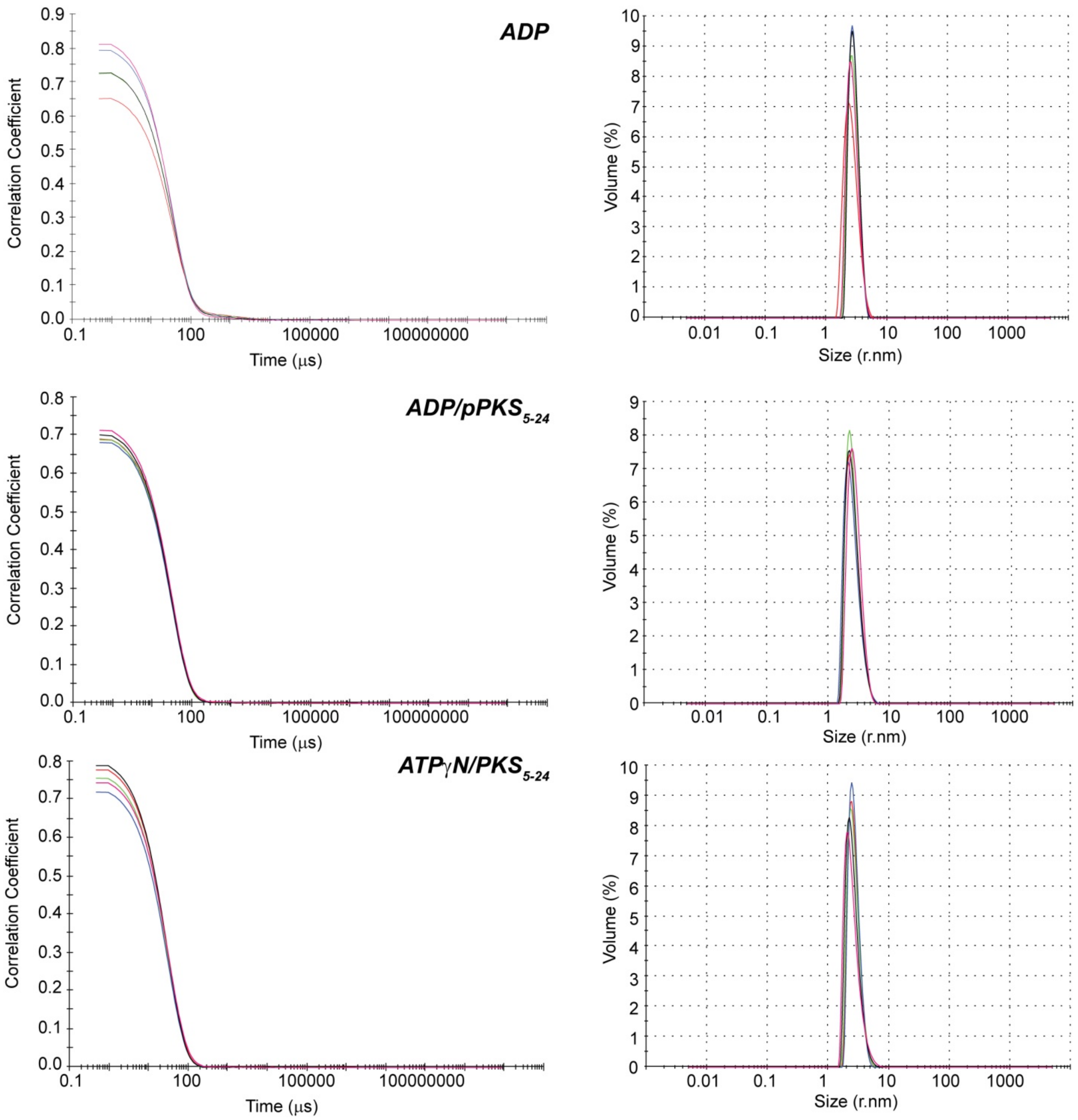

Supplementary Figure 10: The dynamic light scattering data for PKA-C/ADP, PKA-C/ADP/pPKS$_{5-24}$.and PKA-C/ATPγN/PKS$_{5-24}$. The raw scattering correlation plot for all runs as well as the size of the particles from the fit are shown (n=4). Data are reported in the Source Data file.

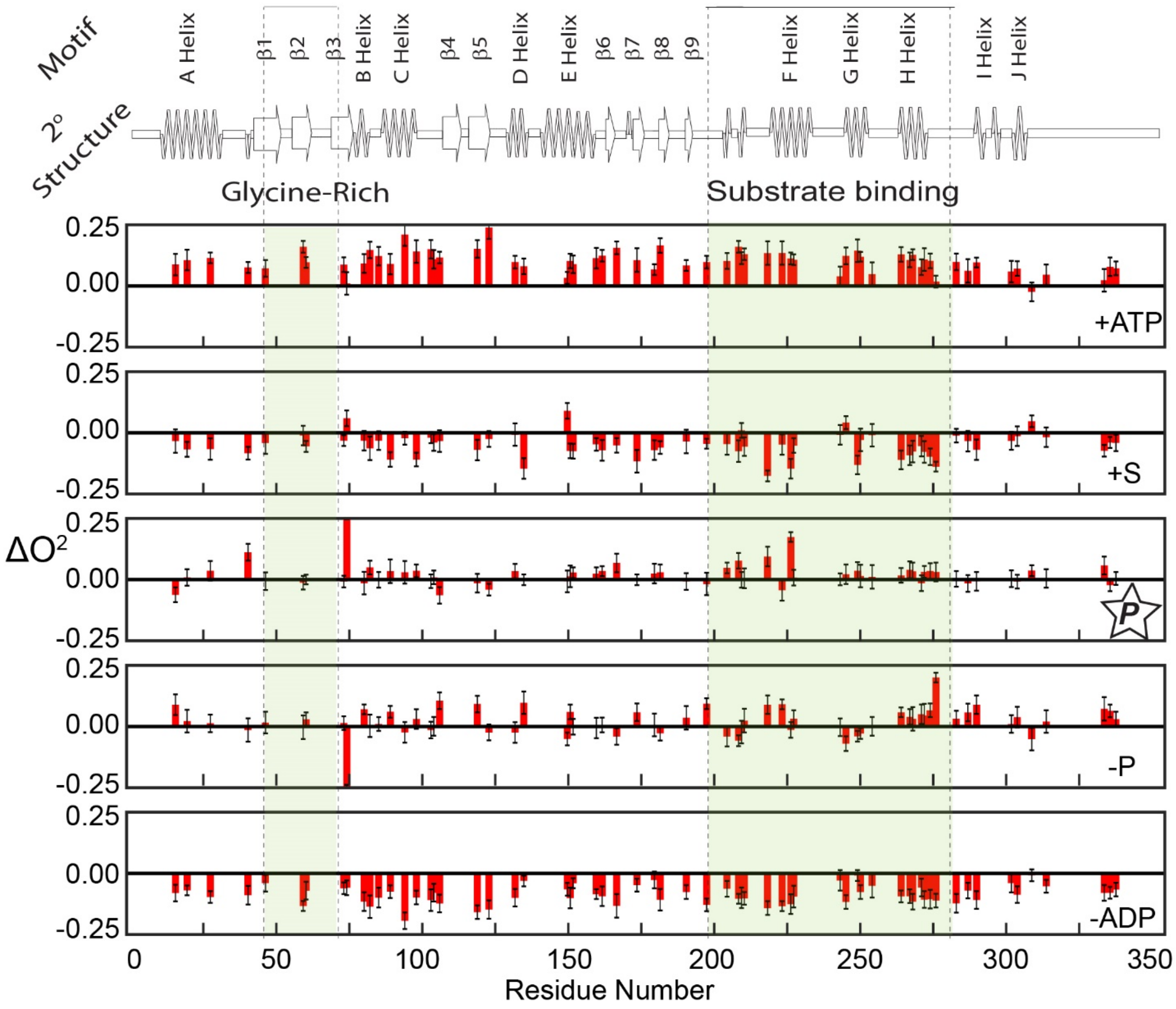

Supplementary Figure 11: Change of Methyl Group Order Parameters (ΔO^2) as a function of residue as measured by coherence violated cross-correlation spectroscopy for each step over the catalytic cycle, with an average difference in ΔO^2 of 0.10, -0.05, 0.02, 0.02, and -0.07 for each step, respectively, where the error bars are derived from the standard deviation of the fitting of the order parameters.

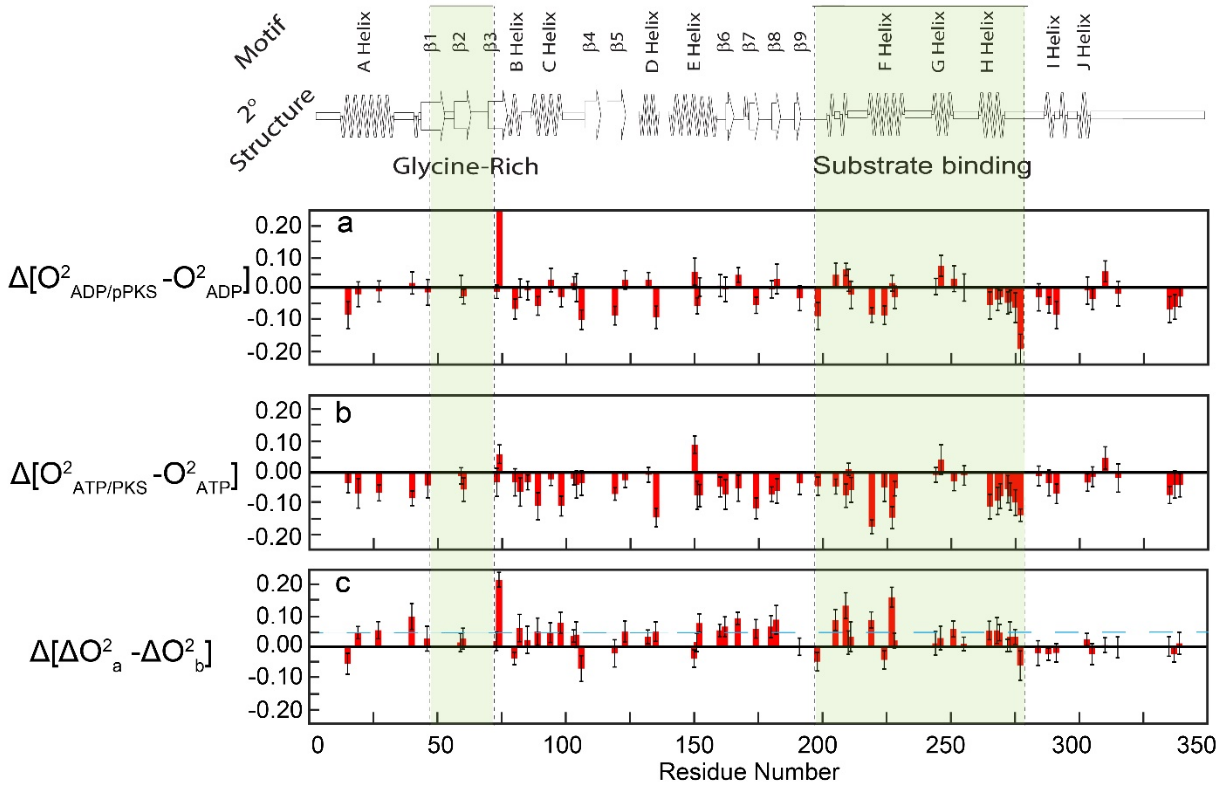

Supplementary Figure 12: Changes in order parameters between the Michaelis-Menton and the product formation complexes along the catalytic cycle. Panel *a* denotes the differences for the methyl group order parameters between PKA-C/ADP and PKA-C/ADP/pPKS complexes; panel *b* shows the corresponding differences between PKA-C/ATP and PKA-C/ATP/PKS complexes. Panel *c* shows the differences in order parameters between panels a and b $\{\Delta\Delta O^2=\Delta[\Delta O^2_a - \Delta O^2_b]\}$. The positive values indicate a rigidification of the methyl groups, highlighting the sign of opposite cooperativity. The dashed blue line indicates a 0.05 cutoff. The error bars are derived from the standard deviation of the fitting of the order parameters.

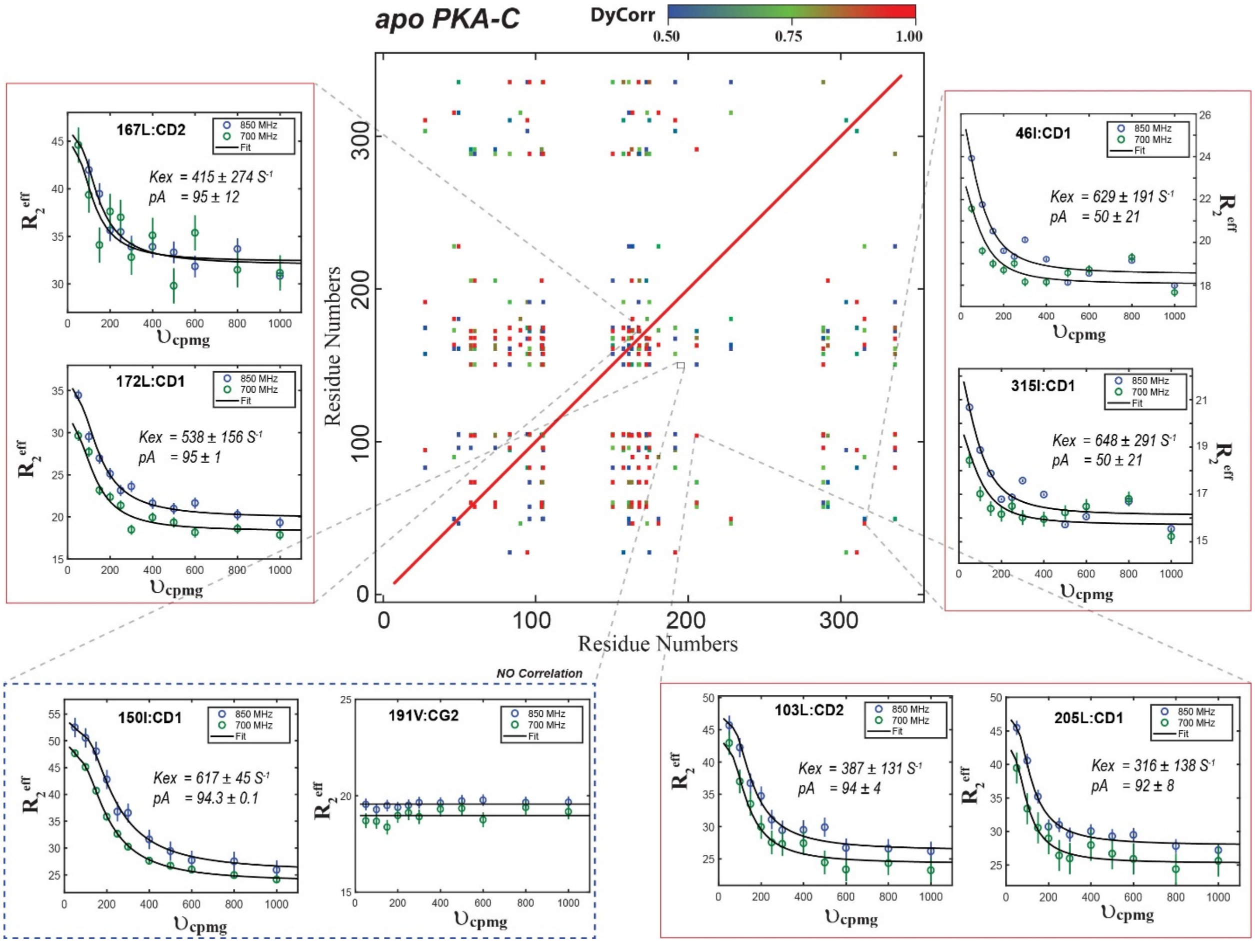

Supplementary Figure 13: Dynamic Correlation (DyCorr) maps of apo state of PKA-C with few representative residues showing correlated methyl dispersion profiles. The error bar in the CPMG profiles were estimated by replicates of the experiments at 50, 200 and 1000 Hz.

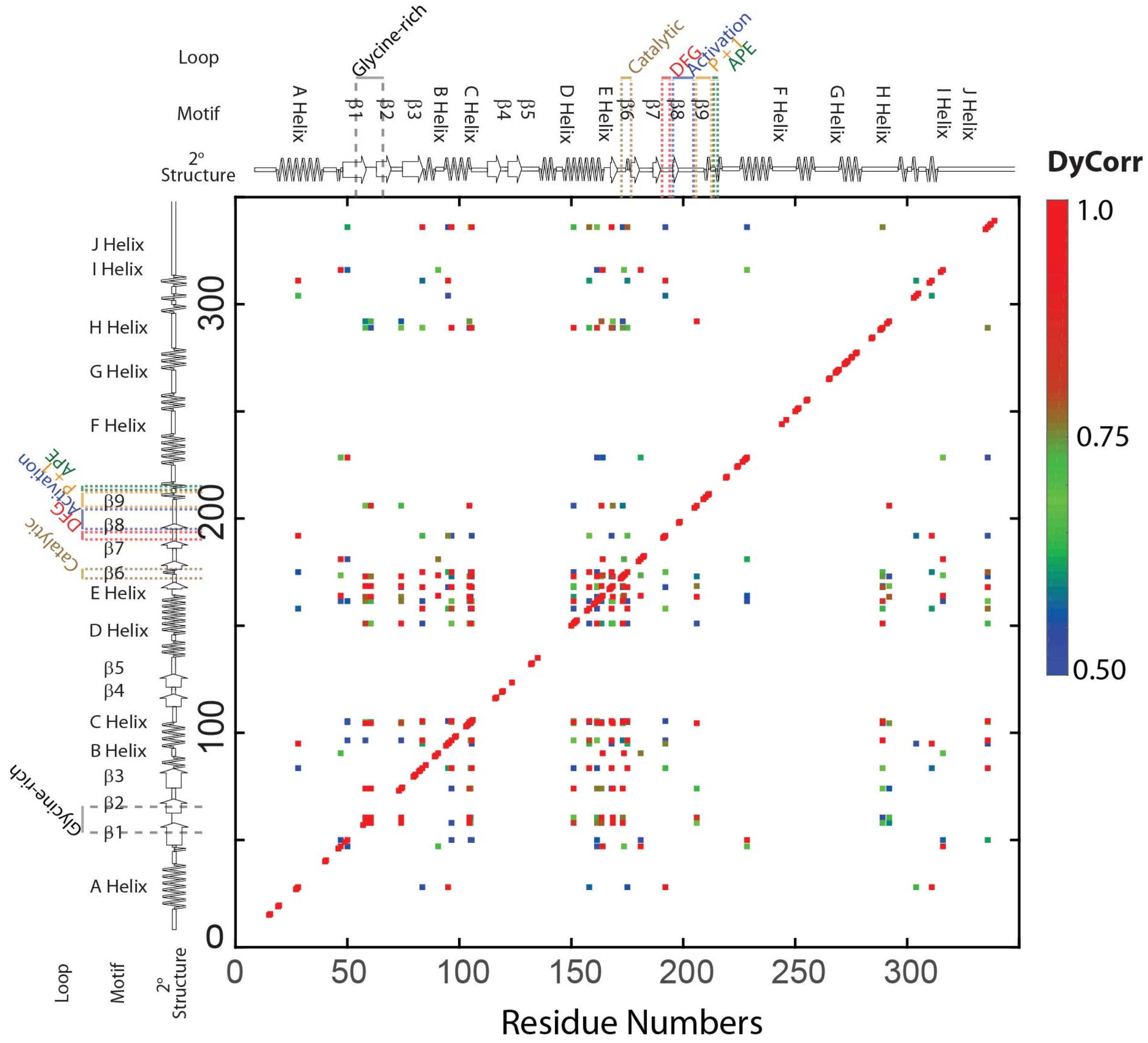

Supplementary Figure 14: Dynamic Correlation maps for the apo state of PKA-C, showing the dynamically uncommitted state for the methyl groups, where the diagonal dots denote the assigned residues.

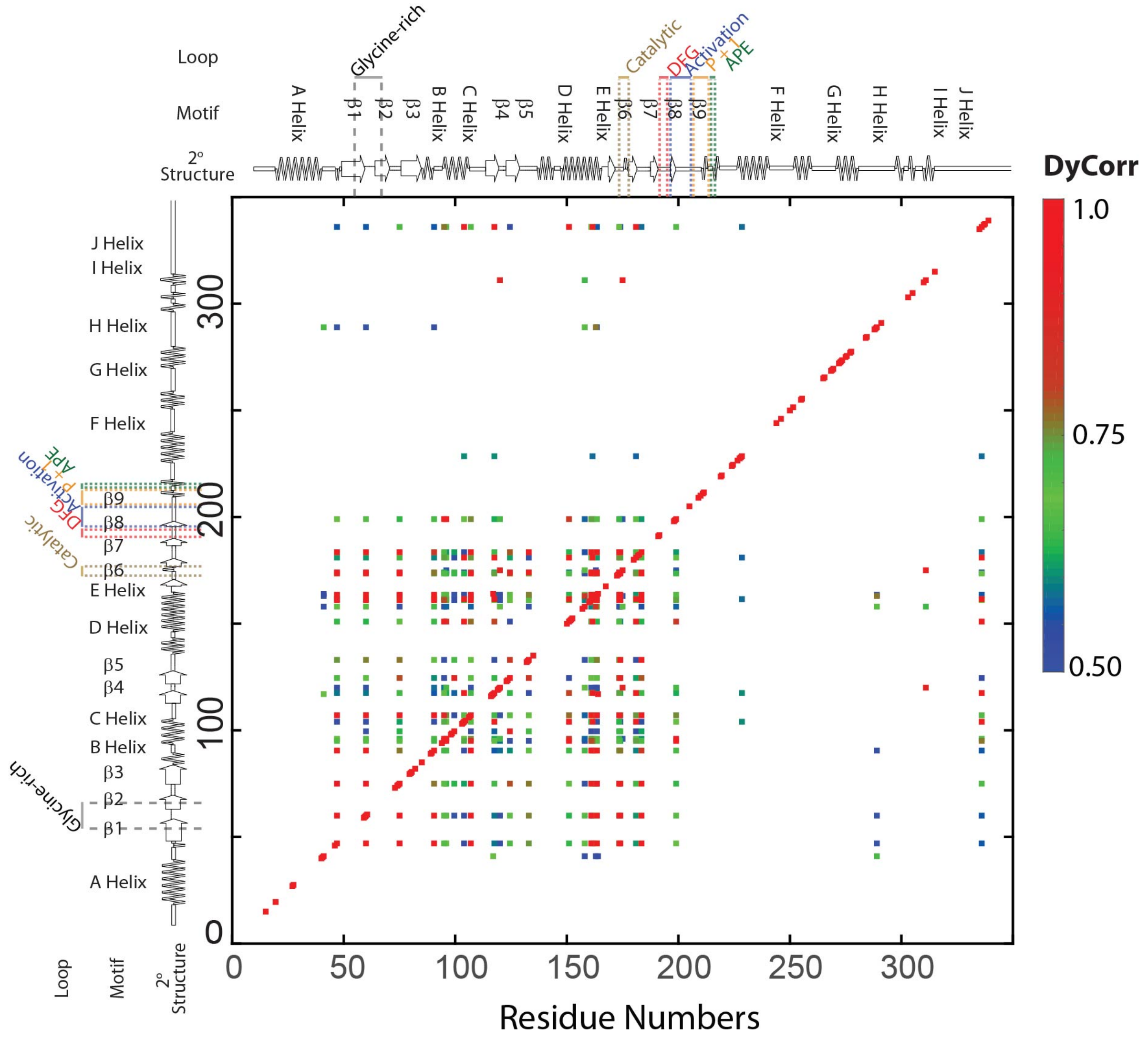

Supplementary Figure 15: Dynamic Correlation maps for the binary state of PKA-C/ATPγN showing the methyl groups that move synchronously throughout the entire kinase.

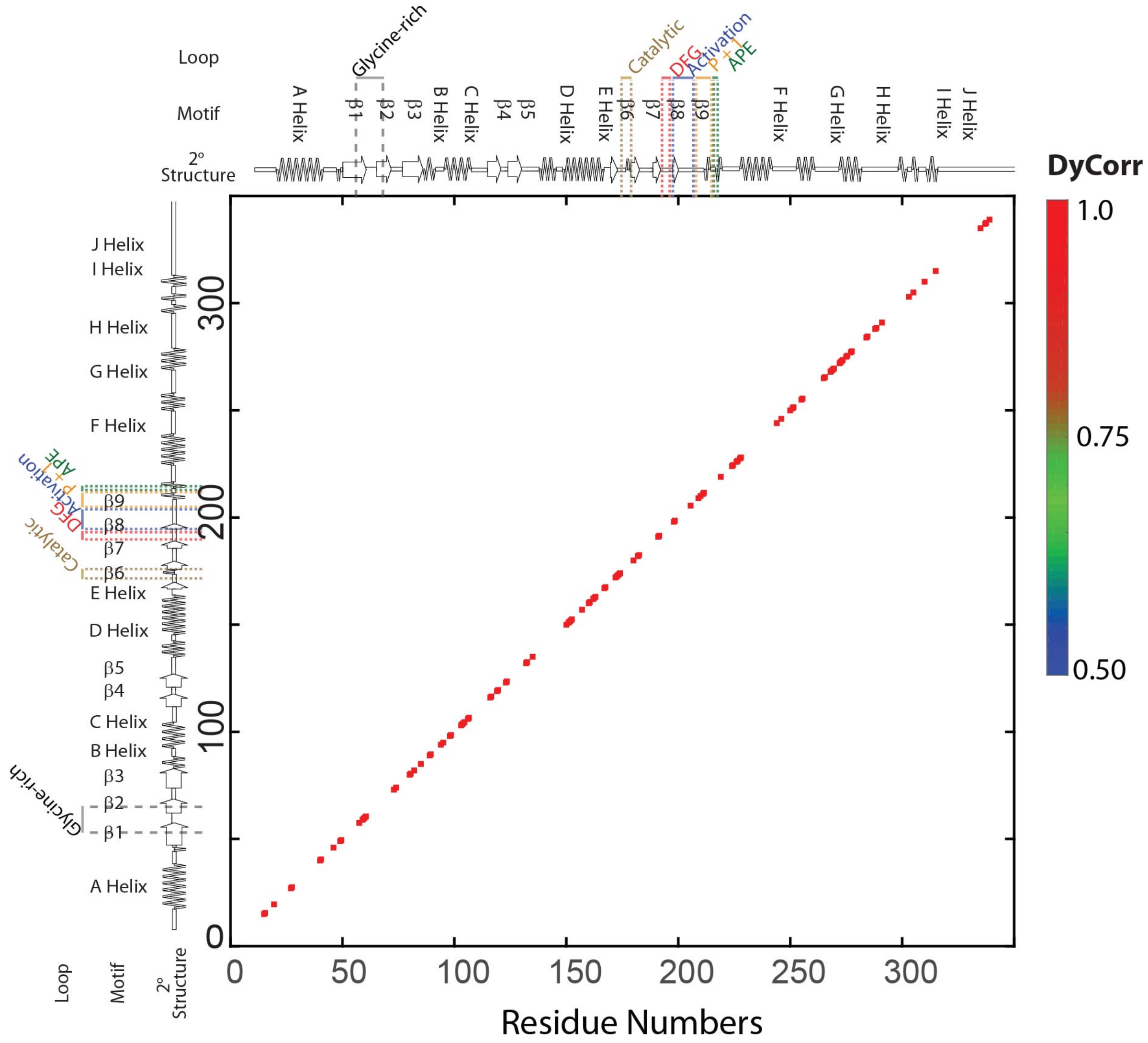

Supplementary Figure 16: Dynamic Correlation maps for the ternary state of PKA-C/ATPγN/PKS$_{5\text{-}24}$ showing the dynamically quenched state.

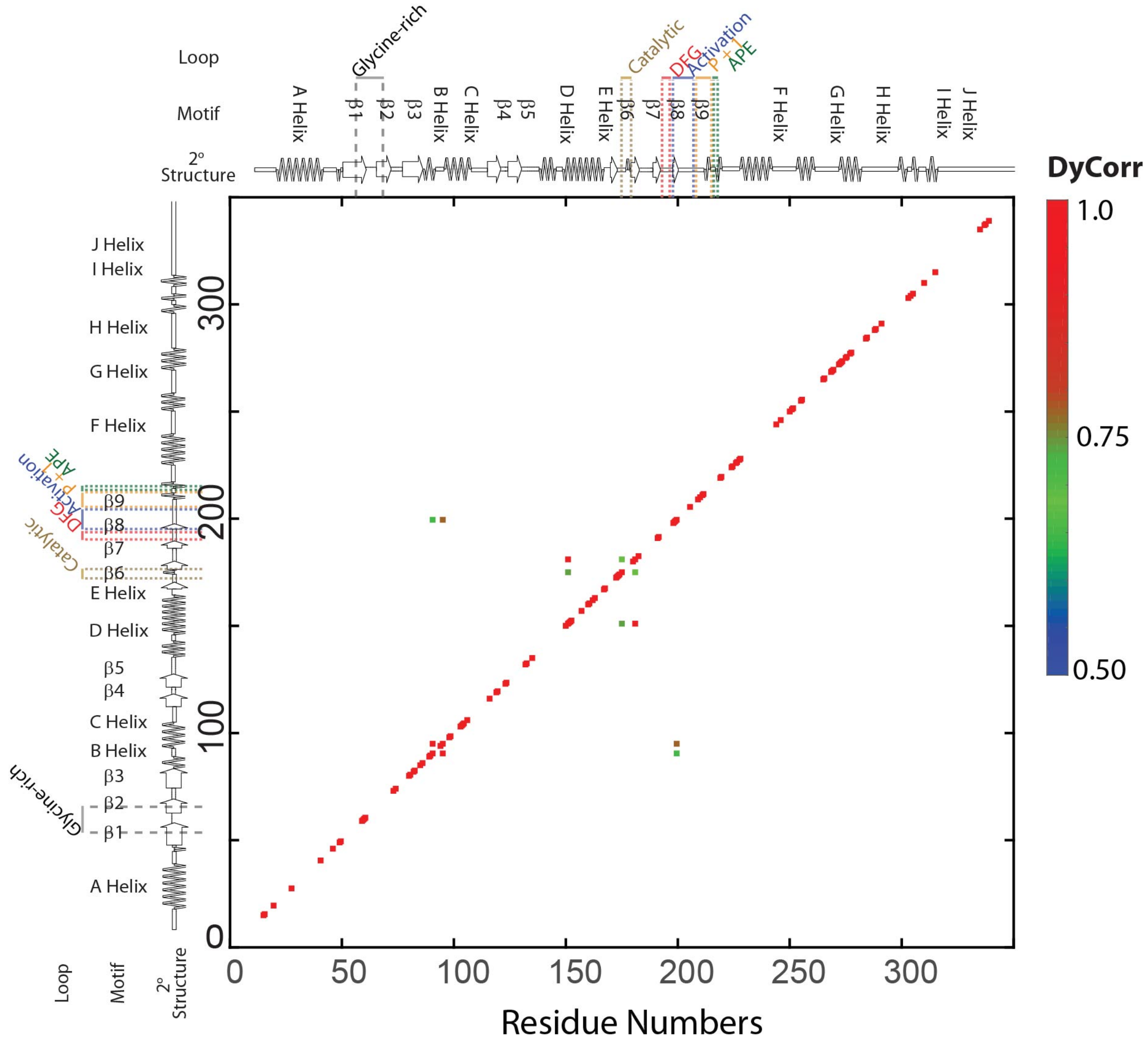

Supplementary Figure 17 Dynamic Correlation maps for the ternary state of PKA-C/ADP/pPKS$_{5\text{-}24}$ showing the sparse emergence of correlated slow dynamics.

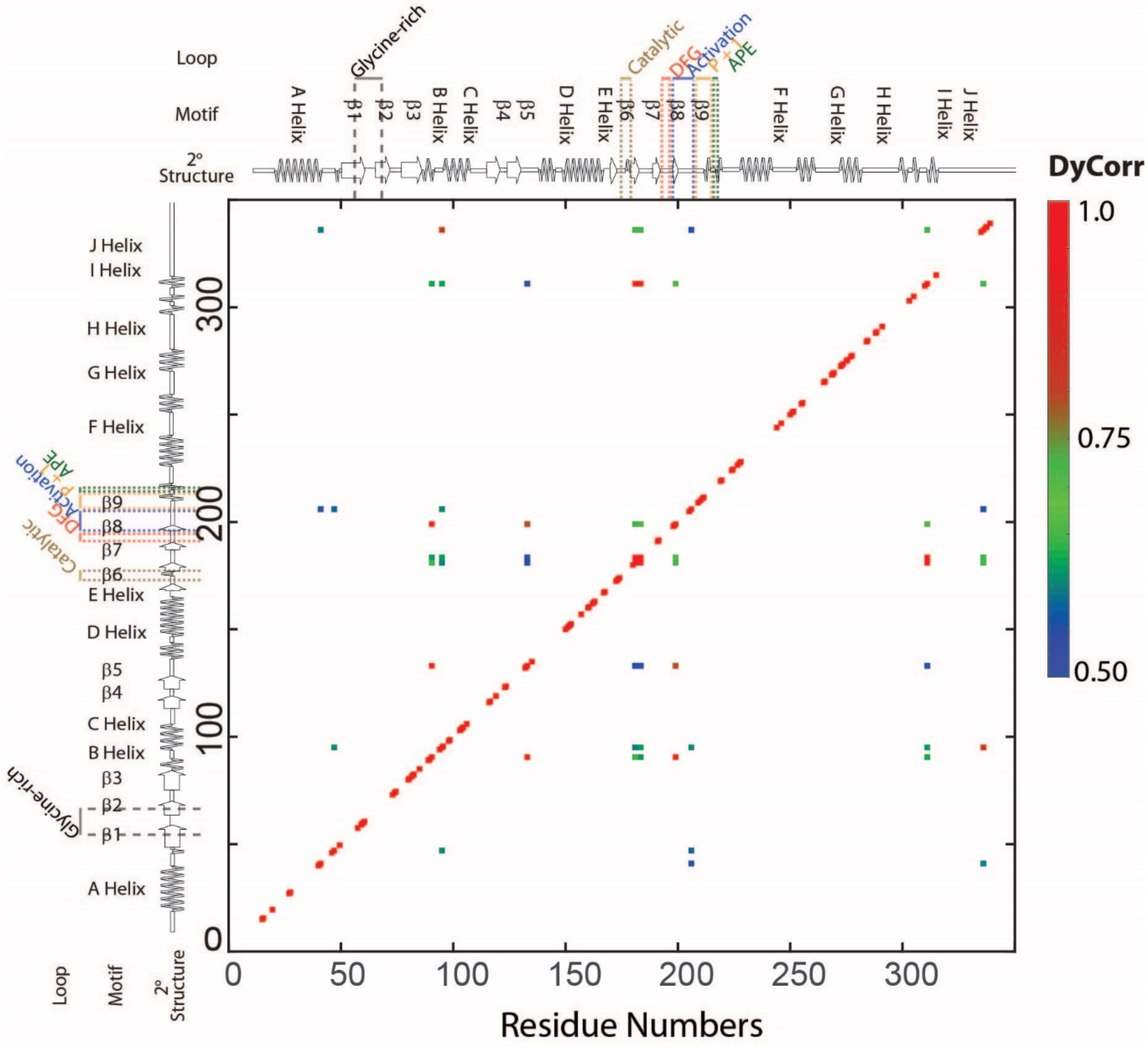

Supplementary Figure 18 Dynamic Correlation maps for the binary state of PKA-C/ADP showing the methyl groups that move asynchronously throughout the entire kinase.

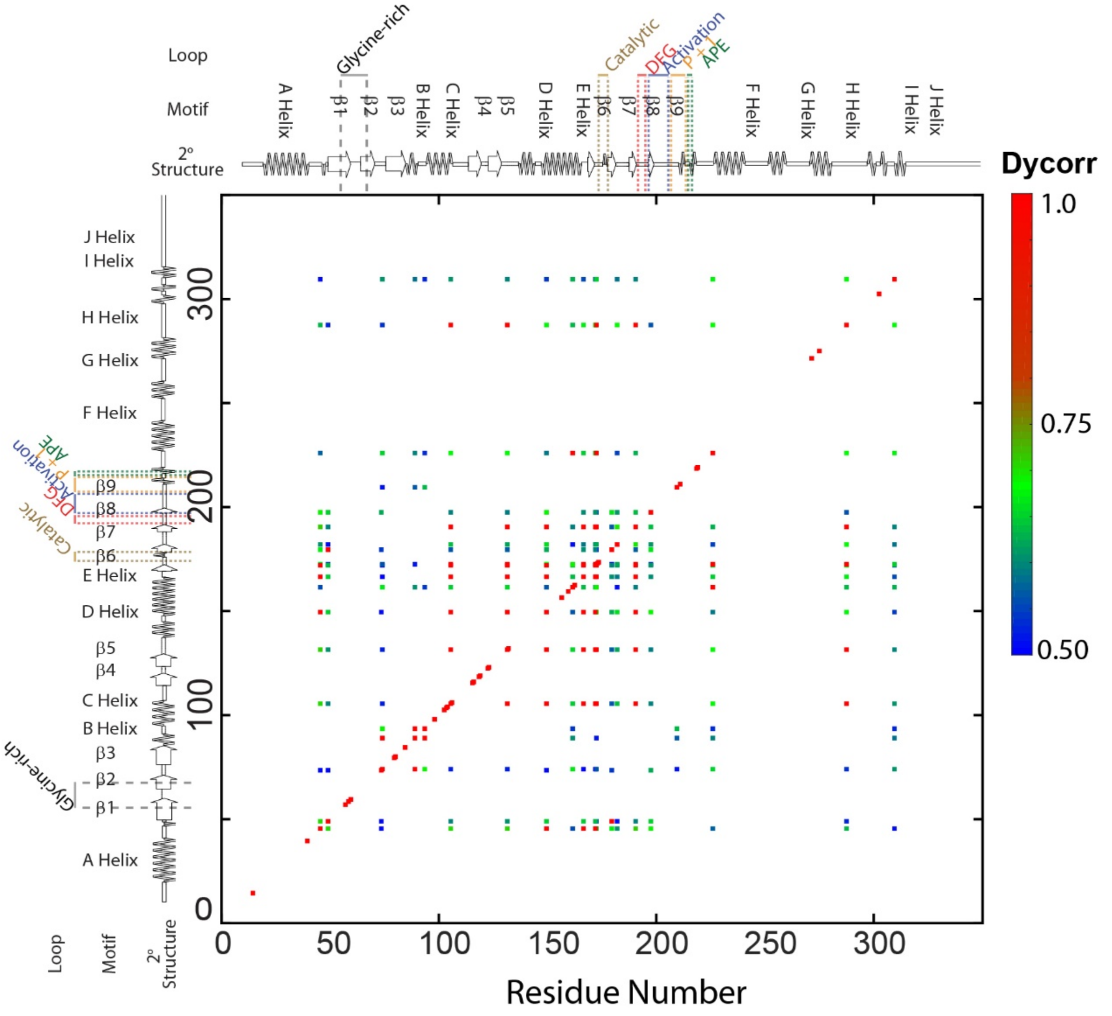

Supplementary Figure 19: Dynamic Correlation maps for the binary state of Y204A/ATPγN showing sparse correlations between the methyl groups, indicating a poorly coordinated response of the mutant enzyme to nucleotide binding compared to WT.

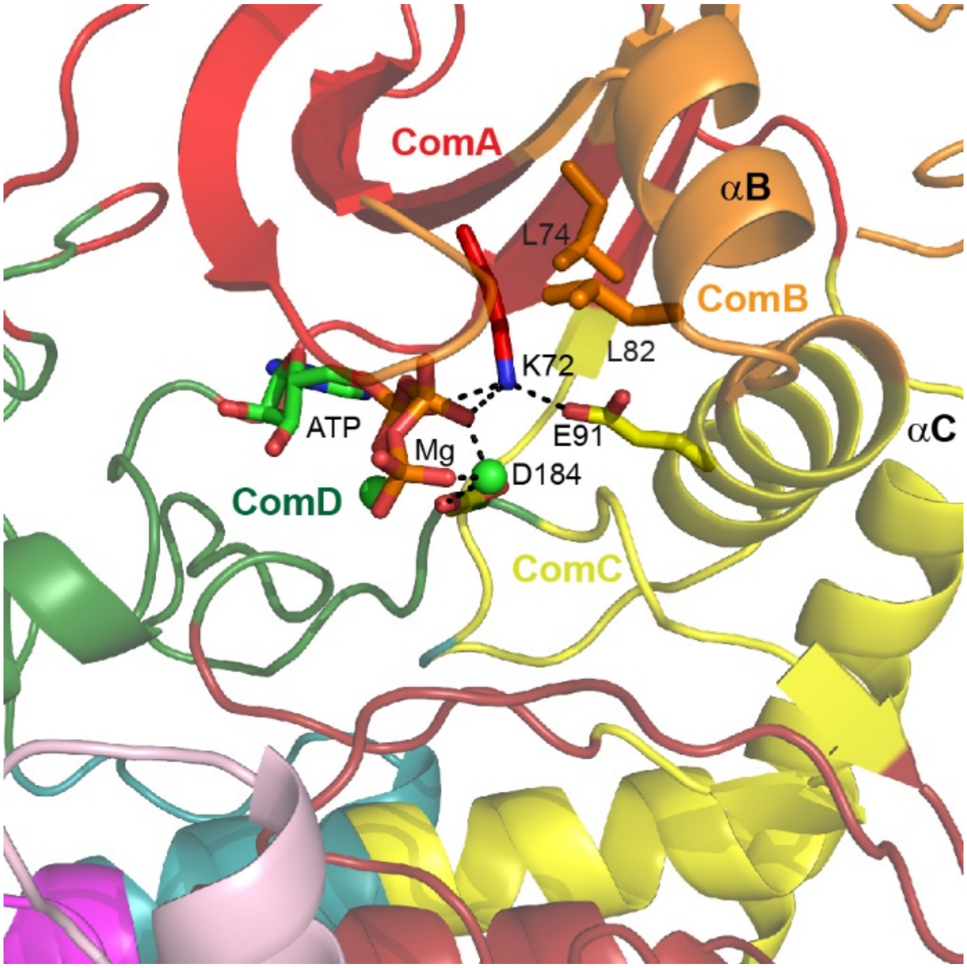

Supplementary Figure 20. The community map analysis of ATP/2Mg shows that extensive electrostatic networks around the Mg and γ-phosphate stabilize the key salt bridge between K72-E91, and that of D184, and bring together the communities of A, B , C and D. The methyl groups of L74 and L82 are probes reflecting the local protein motions in the NMR experiment.

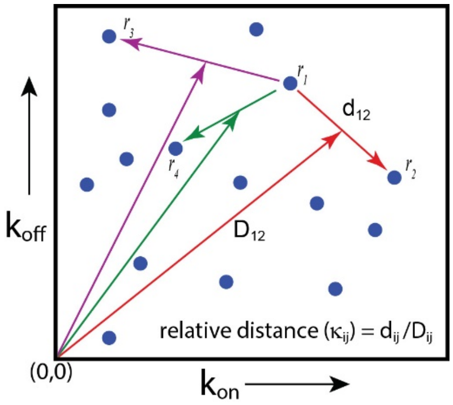

Supplementary Figure 21. Graphical representation of k_{on} and k_{off} in DyCorr. DyCorr is evaluated by calculating the relative distance between the values in the two dimensional space along k_{on} and k_{off}. Every residue which shows chemical exchange is represented by a dot in the k_{on}/k_{off} space.

Supplementary Table 1: PCA and standard deviation of the distribution of states for each of states long the catalytic cycle analyzed by CONCISE analysis.

	PC 1	Standard Deviation
Apo	-1.26 ± 0.09*	0.51 ± 0.04
ATPγN(low Mg^{2+})	-0.58 ± 0.13	0.43 ± 0.03
ATPγN /PLN_{1-19}	0.57 ± 0.12	0.73 ± 0.07
ATPγN /PKI_{5-24}	0.96 ± 0.13	0.52 ± 0.06
ADP	-0.02**	0.72
ADP /$pPKS_{5-24}$	0.74	0.39
ATPγN /PKS_{5-24}	0.67	0.42

*For the ATPγN (low Mg), ATPγN/PLN_{1-19} and ATPγN/PKI_{5-24} states the average and variance between each fit is reported (n=4), and the averages serve as references.

** For the ADP, ADP/$pPKS_{5-24}$, ATPγN/PKS_{5-24} states, a single fit against the above references is reported.

Supplementary Table 2. Entropic changes (cal/K·mol) with respect to apo PKA-C determined from ITC, MD, and NMR data.

	ITC	*NMR*		*MD*
	ΔS_{total}	$\Delta S_{conf}^{protein}$ (model-free analysis)[a]	$\Delta S_{conf}^{protein}$ (model-dependent analysis)[b]	$\Delta S_{conf}^{protein}$
**ATPγN**	10.3±0.7	-29±10	-36±5	-32±2
**ATPγN/PKS**	-28±2	-14±8	-17±3	-25±1
**ADP/pPKS**	-8.0±0.7	-20±9	-24±3	-32±2
**ADP**	5.0±0.7	-25±9	-31±4	NA

[a] In the model-free analysis(1), conformational entropy is determined as the difference of the order parameters of the methyl groups between the two states:

$$\Delta S_{conf,a\to b}^{protein} = s_d N_{\chi}^{prot}\,\Delta\left[\langle O_b^2\rangle - \langle O_a^2\rangle\right],$$

with the coefficient s_d equal to -1.8 $cal/(mol \cdot K)$ and N_{χ}^{prot} representing the total number of torsional angles for methyl-bearing residues.

[b] In the model-dependent analysis(2), conformational entropy is determined via the following equation:

$$\Delta S_{conf,a\to b}^{protein} = -k_B \sum_i ln\left\{\frac{3-\left(1+8O_{b,i}\right)^{1/2}}{3-\left(1+8O_{a,i}\right)^{1/2}}\right\}$$

Supplementary Table 3. Linear methyl side chain resonances reporting on the open to closed transition as identified by CONCISE.

ADP

L27-Cδ1	L40-Cδ2	I46-Cδ1	V60-Cγ1	L74-Cδ1	V80-Cγ1	L82-Cδ2	I85-Cδ1
L89-Cδ2	I94-Cδ1	V98-Cγ2	L103-Cδ1	L106-Cδ1	L116-Cδ2	V119-Cγ1	L132-Cδ1
I135-Cδ1	I150-Cδ1	L157-Cδ1	L160-Cδ2	L162-Cδ1	L162-Cδ2	L167-Cδ1	L172-Cδ2
I180-Cδ1	V182-Cγ2	V191-Cγ1	V191-Cγ2	L205-Cδ1	I209-Cδ1	I210-Cδ1	L227-Cδ2
I244-Cδ1	V251-Cγ1	V251-Cγ2	L265-Cδ1	L269-Cδ2	L272-Cδ2	L273-Cδ2	V275-Cγ2
L277-Cδ1	L284-Cδ1	V288-Cγ1	V288-Cγ2	I291-Cδ1	I305-Cδ1	I315-Cδ1	I335-Cδ1
V337-Cγ1							

ATPγN/High Mg^{2+}

L27-Cδ1	L40-Cδ2	I46-Cδ1	V60-Cγ1	L74-Cδ1	V80-Cγ1	L82-Cδ2	I85-Cδ1
L89-Cδ2	I94-Cδ1	V98-Cγ2	L103-Cδ1	L106-Cδ1	L116-Cδ2	V119-Cγ1	L132-Cδ1
L132-Cδ2	I135-Cδ1	I150-Cδ1	L157-Cδ1	L160-Cδ1	L162-Cδ1	L162-Cδ2	L167-Cδ2
L172-Cδ2	I180-Cδ1	V182-Cγ1	V191-Cγ1	V191-Cγ2	L205-Cδ1	I209-Cδ1	I210-Cδ1
L227-Cδ2	I244-Cδ1	V251-Cγ2	L269-Cδ2	L272-Cδ2	L273-Cδ1	L273-Cδ2	V275-Cγ2

L277-Cδ1	L284-Cδ1	V288-Cγ1	V288-Cγ2	I291-Cδ1	I305-Cδ1	I315-Cδ1	I335-Cδ1
V337-Cγ1							

ADP/pPKS$_{5\text{-}24}$

L40-Cδ2	I46-Cδ1	V60-Cγ1	L74-Cδ1	V80-Cγ1	L82-Cδ1	I85-Cδ1	L89-Cδ2
I94-Cδ1	V98-Cγ2	L103-Cδ1	L106-Cδ1	V119-Cγ1	L116-Cδ2	L132-Cδ1	L132-Cδ2
I135-Cδ1	I150-Cδ1	L157-Cδ1	L160-Cδ1	L162-Cδ1	L167-Cδ1	L167-Cδ2	L172-Cδ2
I180-Cδ1	V182-Cγ2	V191-Cγ1	V191-Cγ2	I209-Cδ1	I210-Cδ1	L227-Cδ2	I244-Cδ1
V251-Cγ1	V251-Cγ2	L265-Cδ1	L269-Cδ2	L272-Cδ2	L273-Cδ2	V275-Cγ2	L277-Cδ1
L284-Cδ1	V288-Cγ1	V288-Cγ2	I291-Cδ1	I305-Cδ1	I315-Cδ1	I335-Cδ1	V337-Cγ1

ATPγN/PKS$_{5\text{-}24}$

L27-Cδ1	I46-Cδ1	V60-Cγ1	L74-Cδ1	V80-Cγ1	L82-Cδ1	I85-Cδ1	L89-Cδ2
I94-Cδ1	V98-Cγ2	L103-Cδ1	L106-Cδ1	L116-Cδ2	V119-Cγ1	L132-Cδ1	L132-Cδ2
I135-Cδ1	I150-Cδ1	L157-Cδ1	L160-Cδ1	L162-Cδ1	L162-Cδ2	L167-Cδ1	L167-Cδ2
L172-Cδ2	I180-Cδ1	V182-Cγ2	V191-Cγ1	V191-Cγ2	I209-Cδ1	I210-Cδ1	L227-Cδ2
I244-Cδ1	V251-Cγ1	V251-Cγ2	L265-Cδ1	L269-Cδ2	L272-Cδ2	L273-Cδ2	V275-Cγ2
L277-Cδ1	L284-Cδ1	V288-Cγ1	V288-Cγ2	I291-Cδ1	I305-Cδ1	I315-Cδ1	I335-Cδ1
V337-Cγ1							

Supplementary Table 4. Single quantum individual site fits of CPMG curves at 700 MHz and 850 MHZ of the ATPγN bound state of PKA-C.

ATPγN	R_{ex} (700 MHz, s^{-1})	R_{ex} (850 MHz, s^{-1})	Δω (ppm)	p_A (%)	k_{ex} (s^{-1})
L40-Cδ1	13.3 ± 0.6	19.8 ± 1.0	0.2 ± 0.1	50.0 ± 16.4	810 ± 88
I46-Cδ1	18.0 ± 0.6	26.7 ± 1.2	0.4 ± 0.2	65.6 ± 18.2	2087 ± 280
L49-Cδ2	21.5 ± 13.3	31.8 ± 19.6	0.5 ± 1.5	61.5 ± 21.9	4032 ± 3867
L59-Cδ1	8.7 ± 0.9	12.8 ± 1.3	0.3 ± 0.4	64.7 ± 20.7	2116 ± 554
I73-Cδ1	3.2 ± 10.2	4.8 ± 11.1	0.1 ± 0.4	50.0 ± 20.2	269 ± 329
L74-Cδ1	19.7 ± 1.6	29.2 ± 2.9	0.4 ± 0.4	65.6 ± 20.0	2635 ± 768
V79-Cγ2	2.4 ± 0.3	2.4 ± 0.3	3.0 ± 0.4	97.6 ± 2.2	100 ± 542
V80-Cγ1	7.2 ± 1.2	7.2 ± 1.2	4.9 ± 0.2	75.8 ± 6.2	24 ± 53
L82-Cδ1	2.5 ± 0.6	3.7 ± 1.0	0.2 ± 0.7	85.2 ± 19.6	1683 ± 1652
I85-Cδ1	5.6 ± 0.3	5.7 ± 0.4	1.2 ± 0.1	94.2 ± 4.5	96 ± 183
L89-Cδ2	7.7 ± 0.5	11.3 ± 0.8	0.2 ± 0.3	68.9 ± 20.5	1918 ± 314
I94-Cδ1	9.5 ± 0.4	13.8 ± 0.6	0.4 ± 0.2	90.2 ± 19.0	1894 ± 294
L95-Cδ1	45.4 ± 3.7	54.0 ± 9.8	1.5 ± 0.4	94.8 ± 12.1	1654 ± 824
V98-Cγ2	6.9 ± 4.2	10.2 ± 5.6	0.3 ± 1.4	50.0 ± 21.2	4087 ± 2811
L103-Cδ1	5.9 ± 0.9	7.8 ± 1.8	1.3 ± 0.7	99.0 ± 20.1	2433 ± 1260
V104-Cγ2	2.0 ± 1.8	2.9 ± 2.2	0.2 ± 1.8	50.0 ± 22.1	3896 ± 3371
L106-Cδ1	11.2 ± 0.5	16.6 ± 0.8	0.3 ± 0.3	73.3 ± 20.1	2296 ± 313
L116-Cδ1	4.1 ± 3.1	6.1 ± 3.8	0.1 ± 0.3	50.0 ± 20.7	530 ± 376
L116-Cδ2	5.3 ± 2.9	7.9 ± 3.9	0.3 ± 1.2	81.3 ± 21.1	3043 ± 2181
V119-Cγ1	5.4 ± 0.4	6.7 ± 0.9	0.8 ± 0.3	98.7 ± 11.7	997 ± 458
V119-Cγ2	2.0 ± 1.2	2.2 ± 1.5	1.7 ± 1.1	99.8 ± 12.0	1151 ± 2349
V123-Cγ2	9.1 ± 2.3	13.5 ± 3.1	0.3 ± 0.6	54.2 ± 19.6	3254 ± 1271
L132-Cδ1	28.1 ± 1.2	41.6 ± 2.6	0.5 ± 0.3	50.4 ± 19.1	2549 ± 394
L132-Cδ2	8.2 ± 3.2	8.8 ± 5.8	0.4 ± 0.2	94.9 ± 15.6	201 ± 162

I135-Cδ1	3.1 ± 1.2	4.1 ± 1.7	1.2 ± 1.0	99.5 ± 14.9	2052 ± 1540
I150-Cδ1	34.2 ± 1.5	46.6 ± 3.9	1.0 ± 0.3	92.6 ± 15.2	2181 ± 427
L157-Cδ1	9.1 ± 0.6	13.5 ± 1.1	0.2 ± 0.2	68.9 ± 20.4	1325 ± 256
L160-Cδ1	4.5 ± 0.6	6.6 ± 1	0.2 ± 0.4	71.4 ± 20.0	2266 ± 1135
L160-Cδ2	7.4 ± 0.5	9.7 ± 0.8	1.3 ± 0.4	98.8 ± 11.3	2353 ± 654
L162-Cδ1	36.0 ± 3.4	53.8 ± 6.9	0.4 ± 0.3	56.4 ± 19.3	1557 ± 586
L162-Cδ2	8.7 ± 0.7	12.8 ± 1.1	0.3 ± 0.4	64.7 ± 20.1	2116 ± 519
I163-Cδ1	11.9 ± 1.9	17.8 ± 3.3	0.1 ± 0.1	50.0 ± 16.9	422 ± 72
L167-Cδ1	2.5 ± 3.2	3.7 ± 3.6	0.2 ± 2.1	74.2 ± 22.6	3254 ± 3974
L172-Cδ2	24.2 ± 0.7	35.8 ± 1.2	0.5 ± 0.2	66.5 ± 18.3	2685 ± 272
L173-Cδ1	19.0 ± 0.9	28.1 ± 2.2	0.4 ± 0.3	58.8 ± 19.8	2565 ± 466
L173-Cδ2	3.3 ± 2.1	3.3 ± 2.8	1.2 ± 0.9	88.3 ± 14.7	25 ± 2720
I174-Cδ1	34.9 ± 1.5	43.4 ± 2.9	0.7 ± 0.1	90.8 ± 9.9	863 ± 223
I180-Cδ1	59.8 ± 4.7	85.7 ± 12.9	1.0 ± 0.5	83.8 ± 19.7	2805 ± 1032
V182-Cγ2	12.5 ± 0.5	18.5 ± 0.8	0.3 ± 0.2	61.2 ± 19.3	2493 ± 282
V191-Cγ1	5.4 ± 0.2	8.0 ± 0.2	0.2 ± 0.2	65.7 ± 19.6	2703 ± 225
L198-Cδ1	45.4 ± 3.7	54.0 ± 8.9	1.5 ± 0.4	94.8 ± 11.6	1654 ± 722
L205-Cδ1	12.4 ± 4.4	16.7 ± 5.5	3.9 ± 2.0	99.3 ± 12.2	8445 ± 2915
V226-Cγ2	2.6 ± 1.1	2.8 ± 1.5	2.2 ± 0.9	99.8 ± 6.0	1428 ± 1653
L227-Cδ2	11.0 ± 6.7	11.9 ± 6.7	6.6 ± 1.5	99.6 ± 1.4	4144 ± 2105
I246-Cδ1	3.4 ± 2.3	5.0 ± 3.4	0.2 ± 0.8	50.0 ± 19.9	4889 ± 4676
V255-Cγ2	2.3 ± 1.9	2.4 ± 2.0	5.0 ± 1.4	99.9 ± 0.9	2409 ± 1913
V288-Cγ1	2.3 ± 0.1	3.4 ± 0.2	0.1 ± 0.2	50.0 ± 20.6	1182 ± 224
I303-Cδ1	3.0 ± 0.6	3.0 ± 0.7	1.9 ± 0.4	93.6 ± 6.4	46 ± 743
V310-Cγ1	9.0 ± 0.3	10.9 ± 0.5	0.7 ± 0.1	97.6 ± 0.5	794 ± 151
I315-Cδ1	2.6 ± 1.2	3.8 ± 2.0	0.1 ± 0.5	77.6 ± 20.4	1275 ± 2589
I335-Cδ1	50.1 ± 3.2	64.4 ± 7.3	1.6 ± 0.4	94.1 ± 15.6	2469 ± 969

Supplementary Table 5: Single quantum individual site fits of CPMG curves at 700 MHz and 850 MHZ of the ADP/ $pPKS_{5-24}$ bound state of PKA-C.

ADP/$pPKS_{5-24}$	R_{ex} (700 MHz, s^{-1})	R_{ex} (850 MHz, s^{-1})	$\Delta\omega$ (ppm)	p_A (%)	k_{ex} (s^{-1})
L46-Cδ1	2.5 ± 3	3.7 ± 3.3	0.07±0.58	50.0± 19.6	590 ± 590
L49-Cδ2	12.3 ± 1.5	13.0± 2.5	0.58± 0.18	94.7± 14.6	280 ± 210
L73-Cδ1	2.0 ± 1.2	2.1 ± 1.6	1.87± 1.00	99.8± 8.7	990 ± 2460
V80-Cγ1	17.0 ± 1.5	19.0 ± 3.4	0.91± 0.33	96.4± 16.7	690± 600
V80-Cγ2	3.5 ± 0.7	10.6 ± 4.4	1.50± 0.86	98.4± 16	64± 250
I85-Cδ1	3.8 ± 0.3	3.6 ± 0.3	1.20± 0.11	82.6± 3.9	17± 7.5
I94-Cδ1	9.4 ± 0.6	10.7 ± 3.0	0.13± 0.10	50.0± 19.6	730± 100
L106-Cδ2	7.5 ± 2.3	7.6 ± 4.0	0.52± 0.19	81.9± 14.0	38± 130
V123-Cγ1	5.1 ± 1.0	5.5 ± 1.7	0.65± 0.37	98.3± 18.3	380± 440
I150-Cδ1	10.2 ± 0.8	15.1 ± 1.4	0.32± 0.39	71.0± 20.0	2170± 620
I174-Cδ1	7.0 ± 0.5	10.1 ± 0.9	0.51± 0.42	94.5± 20.5	2200± 660
I180-Cδ1	7.7 ± 0.6	11.4 ± 1.0	0.25± 0.33	70.3± 20.0	2080± 480
L198-Cδ1	17.5 ± 1.1	22.3 ± 2.7	0.37± 0.09	91.1± 12.2	520±110
L198-Cδ2	4.1 ± 0.7	6.1 ± 1.3	0.11± 0.14	50.0 ± 21.5	980± 370
I246-Cδ1	6.3 ± 4.8	8.8 ± 6.2	1.15± 1.29	98.5± 22.2	3160± 3410
I303-Cδ1	8.6 ± 0.4	12.7 ± 5.1	0.38± 0.59	50.0± 20.3	5160± 2760
I305-Cδ1	2.2 ± 1.9	3.2 ± 2.4	0.87± 1.44	99.2± 18.8	3070± 3330

Supplementary Table 6: Single quantum individual site fits of CPMG curves at 700 MHz and 850 MHz of the ADP bound state of PKA-C.

ADP	R_{ex} (700 MHz, s^{-1})	R_{ex} (850 MHz, s^{-1})	$\Delta\omega$ (ppm)	p_A (%)	k_{ex} (s^{-1})
L40-Cδ1	11.1 ± 0.6	16.4 ± 0.9	0.30±0.24	50.0± 18.2	2400 ± 350
I46-Cδ1	4.1 ± 2.8	6.0± 3.5	0.82 ± 1.41	97.8± 17.7	3900 ± 2400
V80-Cγ1	5.5 ± 2.6	6.2 ± 3.4	2.14± 0.90	99.5± 5.0	1700± 2000
V80-Cγ2	3.2 ± 0.2	4.7 ± 0.5	0.09± 0.16	50.0± 22	780± 160
I85-Cδ1	3.8 ± 0.3	3.6 ± 0.3	1.20± 0.11	99.5± 10.8	1500± 71900
L89-Cδ2	4.9 ± 0.4	5.5 ± 0.9	1.16± 0.29	99.1± 7.5	830± 620
I94-Cδ1	3.9 ± 0.4	5.7 ± 0.6	0.29± 0.51	89.1± 20.5	2600± 750
V104-Cγ2	4.2 ± 3.7	4.8 ± 4.3	2.43± 1.52	99.7±12.8	2200± 3300
L106-Cδ1	4.1 ± 3.0	4.9 ± 3.7	1.51± 1.25	99.5± 10.5	1600± 2200
L116-Cδ2	6.6 ± 3.7	9.5 ± 5.3	0.76± 0.96	96.4± 21.3	3500± 3300
L132-Cδ1	13.7 ± 1.1	15.6 ± 2.4	0.89± 0.23	97.1± 8.5	730± 370
L173-Cδ1	11.3 ±6.6	14.3 ± 9.4	1.9 ± 1.20	98.9 ± 17.1	2800 ± 3600
I180-Cδ1	34.4 ± 2.2	43.9 ± 6.1	0.9 ± 0.27	93.3 ± 11.7	1400 ± 460
V182-Cγ2	6.3 ± 0.7	8.3 ± 1.4	0.80 ±0.42	98.4 ± 19	1400 ± 630
V191-Cγ1	2.5 ± 0.2	2.6 ± 0.3	1.03 ±0.28	99.4 ± 10.7	490 ± 440
L198-Cδ1	20.2 ± 1.2	23.7 ± 2.6	0.99 ±0.28	96.3 ± 6.8	970 ± 400
L205-Cδ1	14.3 ± 4.5	21.1 ± 6.9	0.47 ±0.53	62 ± 20	4400 ± 2200
V226-Cγ2	7.3 ± 1.7	7.3 ± 3.1	0.59 ±0.27	93.5 ± 16.3	110 ± 240
V310-Cγ1	10.7 ± 0.3	13.6 ± 0.6	0.92 ±0.15	97.7 ± 2.3	1400 ± 250
I335-Cδ1	10.7 ± 0.7	15.7 ± 1.3	0.36 ±0.34	80.3 ± 20	2200 ±510

Supplementary References

1. Kasinath V, Sharp KA, & Wand AJ (2013) Microscopic insights into the NMR relaxation-based protein conformational entropy meter. *J Am Chem Soc* 135(40):15092-15100.
2. Yang D & Kay LE (1996) Contributions to Conformational Entropy Arising from Bond Vector Fluctuations Measured from NMR-Derived Order Parameters: Application to Protein Folding. *Journal of Molecular Biology* 263(2):369-382.
