# Supplementary material for: Globally correlated conformational entropy underlies positive and negative cooperativity in a kinase’s enzymatic cycle
Source: Nat Commun. 2019 Feb 18;10:799. doi: 10.1038/s41467-019-08655-7 (PMC6379427; doi:10.1038/s41467-019-08655-7)
Supplement: Supplementary file 3 — Description of Additional Supplementary Information [file 41467_2019_8655_MOESM3_ESM.pdf]

Description of Additional Supplementary Files

File Name: Supplementary Data 1
Description: Methyl order parameter values

File Name: Supplementary Data 2
Description: CHESCA map values

File Name: Supplementary Data 3
Description: DyCorr values
